# COVID-19 patient transcriptomic and genomic profiling reveals comorbidity interactions with psychiatric disorders

**DOI:** 10.1038/s41398-020-01151-3

**Published:** 2021-03-15

**Authors:** Mohammad Ali Moni, Ping-I Lin, Julian M. W. Quinn, Valsamma Eapen

**Affiliations:** 1 Faculty of Medicine, School of Psychiatry, University of New South Wales, Sydney, NSW 2052 Australia; 2 South Western Sydney Area Health Service, Sydney, NSW 2170 Australia; 3 The Garvan Institute of Medical Research, Healthy Ageing Theme, Darlinghurst, NSW 2010 Australia; 4 Division of Surgery and Anesthesia, Royal North Shore Hospital SERT Institute, St Leonards, NSW 2065 Australia

**Keywords:** Medical genetics, Human behaviour, Bipolar disorder, Schizophrenia

## Abstract

Psychiatric symptoms are seen in some COVID-19 patients, as direct or indirect sequelae, but it is unclear whether SARS-CoV-2 infection interacts with underlying neuronal or psychiatric susceptibilities. Such interactions might arise from COVID-19 immune responses, from infection of neurons themselves or may reflect social-psychological causes. To clarify this we sought the key gene expression pathways altered in COVID-19 also affected in bipolar disorder, post-traumatic stress disorder (PTSD) and schizophrenia, since this may identify pathways of interaction that could be treatment targets. We performed large scale comparisons of whole transcriptome data and immune factor transcript data in peripheral blood mononuclear cells (PBMC) from COVID-19 patients and patients with psychiatric disorders. We also analysed genome-wide association study (GWAS) data for symptomatic COVID-19 patients, comparing GWAS and whole-genome sequence data from patients with bipolar disorder, PTSD and schizophrenia patients. These studies revealed altered signalling and ontology pathways shared by COVID-19 patients and the three psychiatric disorders. Finally, co-expression and network analyses identified gene clusters common to the conditions. COVID-19 patients had peripheral blood immune system profiles that overlapped with those of patients with psychiatric conditions. From the pathways identified, PTSD profiles were the most highly correlated with COVID-19, perhaps consistent with stress-immune system interactions seen in PTSD. We also revealed common inflammatory pathways that may exacerbate psychiatric disorders, which may support the usage of anti-inflammatory medications in these patients. It also highlights the potential clinical application of multi-level dataset studies in difficult-to-treat psychiatric disorders in this COVID-19 pandemic.

## Introduction

By mid-August 2020, the global COVID-19 pandemic due to novel coronavirus SARS-CoV-2 has resulted in over 20 million people infected and three quarters of a million deaths worldwide. The wide distribution of the virus reflects its spread by asymptomatic infected individuals and long incubation period compared to previous epidemic coronaviruses SARS-CoV^1^ and MERS-CoV^2^. Thus far it is contained only by contagion-reducing measures such as quarantine, social distancing and isolation, school and business closures and border control^3^. While vital for public health, these strategies increase psychological stress^4^ which may exacerbate psychiatric disorders in susceptible individuals, but little is known about whether the genomic functional changes in COVID-19 are also involved in biological pathways inherent to these psychiatric disorders. In this regard, it is noteworthy that headache is a common symptom of COVID-19 and other symptoms of central nervous systems (CNS) involvement has also been reported including dizziness, epileptic seizure, confusion, and even stroke^5^.

SARS-CoV-2 infection gives rise to a complex immune response^6^ in which some common virus responses, such as type I interferon production, are unusually low. Severe COVID-19 can trigger a crisis involving very elevated circulating inflammatory factor levels (a ‘cytokine storm’), with disparate parts of the immune system highly activated leading to severe life-threatening sickness^7^. In addition, deleterious viral effects in the cardiovascular and pulmonary systems can interact with existing comorbid conditions^8–11^. There is also evidence that SARS-CoV-2 infects nerve cells^12,13^ which suggests a potential to directly affect the central nervous system (CNS) function. This could also interact with pre-existing neurological conditions. However, it is also likely that there are powerful and rapid COVID-19-CNS interactions mediated by the strong humoral immune responses elicited by the infection, particularly in susceptible individuals. Hence it is important to characterise and understand these interactions and the immune and inflammatory underpinnings that trigger such interactions. Given that it is difficult to study this extremely multifaceted nature of the pathophysiology directly in individuals affected with severe COVID-19, comparing common inflammatory substrates alongside shared signalling and ontology pathways holds promise.

SARS-CoV-2 can enter many cell types, including neurons, by interacting with angiotensin-converting enzyme 2 (ACE2)^14^ and there are plausible pathological mechanisms by which CNS function could be affected. There are documented pathogen-triggered acute psychiatric manifestations, for example, the paediatric autoimmune neuropsychiatric disorder associated with Streptococcus (PANDAS), characterised by sudden-onset severe tics, obsessive-compulsive symptoms and other debilitating neuropsychiatric symptoms^15^. This is thought to be the result of autoimmune antibodies mistakenly targeting basal ganglia antigens instead of the intended bacterial antigen and producing antinuclear antibodies triggering the onset of symptoms. Dysregulated immune responses to infection and resulting neuroinflammation are documented to cause or exacerbate neurological dysfunction^16^. Further, both psychological distress resulting from the COVID-19 pandemic and the stress of being infected may activate the hypothalamic-pituitary-adrenal (HPA) axis and the sympathetic nervous system, with consequent release of glucocorticoids and catecholamines. Glucocorticoids affect many aspects of general physiology and behaviour, and their immunosuppressive effects have been postulated as contributing to PTSD symptoms via the HPA axis changes, which in turn may exacerbate both emotional dysregulation and physical comorbidities^17,18^.

Pathogenic influences on brain functions may also act via other mechanisms, as dysregulations in immune function resulting from neuroendocrine interactions with aspects of the immune systems may unmask predispositions to infectious and inflammatory diseases. In the example of PTSD, resulting in HPA axis dysregulation alongside impaired glucocorticoid signalling can give rise to a pro-inflammatory state that may lead to cardiovascular, respiratory, gastrointestinal, inflammatory and autoimmune diseases^19^. Bipolar disorder and schizophrenia are also linked to immune dysfunction^20,21^ with both disorders carrying a higher risk of developing respiratory diseases^22^. Individuals with schizophrenia and related disorders have already been noted to have increased risks of infection and poorer health outcomes amidst the COVID-19 pandemic^23^.

These lines of evidence suggest that COVID-19 infection may share pathological determinants with some psychiatric disorders through which they may interact. Such interactions can be examined by studying cellular pathways that are altered in these conditions and SARS-CoV-2 infected individuals. To address these issues we applied computational and bioinformatics approaches to study SARS-CoV-2 blood cell and immune panel transcriptome and GWAS data. These studies identified SARS-CoV-2 acute response genes concordant with bipolar disorder, PTSD and schizophrenia. For these genes, we performed extensive functional analyses, and then applied data mining approaches to identify validated biomarkers in order to determine how SARS-CoV-2 response genes and pathways may interact with these three psychiatric disorders.

## Results

**Fig. 1:**
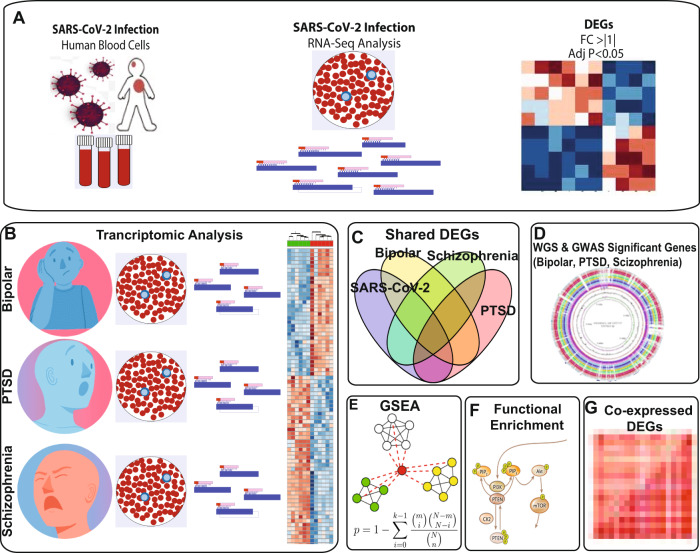
Flow diagram of the study. RNA-Seq, GWAS and WGS analyses define genomic relationships and infection responses between the novel coronavirus SARS-CoV-2, and Bipolar, PTSD and Schizophrenia psychiatric disorders. **A** RNA-Seq gene expression profiling of human peripheral blood mononuclear cells infected with SARS-CoV-2. **B** RNA-Seq gene expression profiling of human peripheral blood mononuclear cells for the bipolar disorder, PTSD and schizophrenia. **C** Sparse overlapping gene expression signature between the SARS-CoV-2 and bipolar disorder, PTSD and schizophrenia. **D** WGS and GWAS data from the different curated data sources and filtered for the bipolar, PTSD and schizophrenia and identified concordant genes with the COVID-19 GWAS biomarker. **E**–**G** A similar conclusion was reached using the functional enrichment, pathways and co-expression analyses of the concordant genes.

We designed a systematic and quantitative analysis framework to investigate the genetic and transcriptomic associations of the COVID-19 with bipolar disorder, PTSD and schizophrenia disorders. All datasets were obtained from publicly available sources of transcriptome, GWAS and WGS data. The flow diagram of the study methodology is shown in Fig. 1. RNA-Seq data was used for transcriptomic analyses, and GWAS and WGS data were used to identify genomic relationships to susceptibility to severe COVID-19 outcomes, as well as to bipolar disorder, PTSD and schizophrenia. RNA-Seq gene expression profiles of PBMC samples from COVID-19 patients are shown in Fig. 1A, and gene expression of PBMCs from bipolar disorder, PTSD and schizophrenia affected individuals are shown in Fig. 1B. Sparse gene expression signatures of SARS-CoV-2 that overlap with those of bipolar disorder, PTSD and schizophrenia disorders are presented in Fig. 1C. Figure 1D depicts WGS and GWAS data analyses from curated data sources, filtered for the psychiatric disorders and genes identified as concordant with the COVID-19 GWAS biomarkers. Genes identified as dysregulated in both COVID-19 and one of the psychiatric disorders were analysed to identify common signalling pathways, gene ontology (GO) and co-expression-networks. Figure 1E–G show similar conclusions that were reached using functional enrichment, pathways and co-expression analyses of the concordant genes.

### Differential gene expression analysis identifies significant PBMC-expressed alteration in both COVID-19 patients and psychiatric disorders

**Fig. 2:**
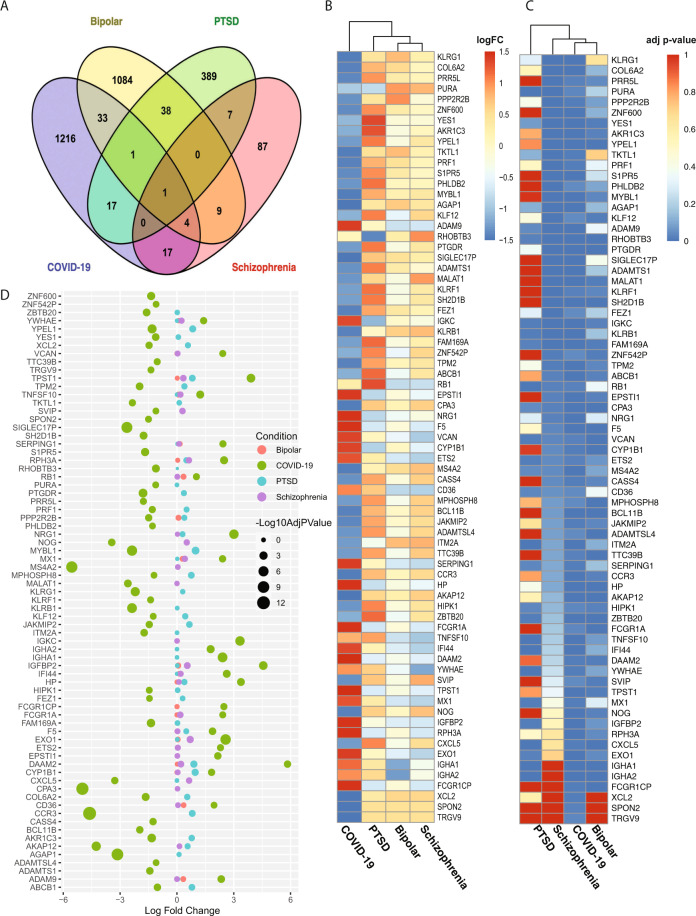
Comparison of RNA-Seq analyses of SARS Cov-2 infected patient whole blood PBMC reveals shared common genes with the Bipolar disorder, PTSD and Schizophrenia PBMC transcriptomic genes. **A** Venn diagram shows the number of common significant genes of SARS Cov-2 with the bipolar disorder, PTSD and schizophrenia. **B** Heat map of the log fold changes for the shared common genes between SARS-CoV-2 and either of the bipolar disorder, PTSD and schizophrenia. **C** Heat map of the adjusted p-values for the shared common genes between SARS-CoV-2 and either of the bipolar disorder, PTSD and schizophrenia. **D** Bubble plot shows the combined log fold changes and adjusted *p*-values for the shared common genes between SARS-CoV-2 and either of the bipolar disorder, PTSD and schizophrenia.

RNA-Seq analyses were used to compare PBMC transcriptome profiles of COVID-19 patients with those of individuals diagnosed with one of the three major psychiatric disorders. PBMC gene expression profiling for COVID-19 patients is shown in Fig. 1A, and comparisons with bipolar disorder, PTSD and schizophrenia are shown in Fig. 2A–D. The latter comparisons revealed a sparse overlapping gene expression signature.

To understand the transcriptional effects of COVID-19 on PBMCs, we identified genes differentially expressed relative to control patients using a stringent cut-off threshold of absolute log_2_ fold change >1, with an adjusted *p*-value < 0.05. We used these candidate genes in further analyses, first comparing the upregulated and downregulated genes with the significant upregulated and downregulated genes of bipolar disorder, PTSD and schizophrenia. The number of common dysregulated genes of COVID-19 patients with psychiatric disorders is presented in the Venn diagram (Fig. 2A). Only a small proportion of genes were changed in expression in a similar direction on both COVID-19 and psychiatric disorders; furthermore, most were differently affected in each condition, as might be expected with such different conditions. Heat map and bubble plot visualisation show the striking nature of the unique transcriptional signature induced upon SARS-CoV-2 infection (Fig. 2B–D). Of the top 1289 COVID-19-response genes, 73 genes were similarly up or downregulated in at least one of the psychiatric disorders. COVID-19 patient PBMC profiles shared 39, 19 and 22 dysregulated genes with those with bipolar disorder, PTSD and schizophrenia, respectively (Fig. 2A–D). However, among these genes altered in COVID-19, two similarly affected genes (VCAN, RHOBTB3) were also observed in bipolar disorder and PTSD, five genes (PTGDR, SH2D1B, AKR1C3, YES1 and RHOBTB3) were shared between bipolar disorder and schizophrenia and one gene (RHOBTB3) is commonly dysregulated among all of three psychiatric conditions and COVID-19. Collectively, these data suggest that although the novel coronavirus SARS-CoV-2 is closely related to the three psychiatric disorders in terms of transcriptomic profiles, the RHOBTB3 gene is the only gene shared by all of these four diseases. The RHOBTB3 gene encodes a member of the evolutionarily conserved RhoBTB subfamily of Rho GTPases that have many intracellular functions, including roles in intracellular transport, and are expressed leucocytes and in the brain tissue of patients with bipolar disorder or schizophrenia^24^.

### Gene ontology and cell signalling pathway analyses identify enriched inflammatory and immune responses to COVID-19 that are also seen in psychiatric disorders

COVID-19 patients can exhibit neurological manifestations^5,25^ consistent with emerging evidence of important interactions between the CNS and immune system^26^. Elevated inflammation is seen in patients with major depression^27^ and inflammatory cytokines alter functions of CNS neurotransmitters which can be reduced by anti-cytokine treatment^28^. There is also strong evidence for similar immune interactions in PTSD and bipolar disorder, notably IL-1β, IL-6 and TNFα (cytokines part of the immune response to pathogens) affecting CNS neurons and circuits and thus neurotransmission, glucocorticoid function, memory, and social behaviours^29^. These cytokines in COVID-19 are released by circulating monocytes in response to disease development, as well as due to monocyte infection by SARS-CoV-2. CNS-immune system interactions involve upstream regulators of these cytokines such as CLOCK, ERK1, GSK3β and P11 genes, which influence increased activity levels and sleep disturbances also seen in bipolar disorder^30^. Schizophrenia also has clear associations with immune dysfunction^31,32^, with a GWAS study implicating the MHC1 locus (containing over 250 immune genes) in schizophrenia incidence^33^.

After establishing the PBMC differential expression profiles common to COVID-19 and the psychiatric disorders, we conducted thorough gene ontology and cell signalling pathway analyses using several curated databases (including The Gene Ontology, WikiPathways, BioCarta, Reactome, and Panther databases) to ascertain how the immune systems of COVID-19 and the psychiatric disorders interact. The top 25 significant signalling pathways and biological process ontology pathways for the bipolar disorder, PTSD and schizophrenia are presented in supplementary Fig. 1A–F, and all the significant pathways and GO are shown in the supplementary Tables 1–6.

### Targeted immune profiling of COVID-19 patient systemic infection immune responses show perturbed cell signalling pathways in common with psychiatric disorders

**Fig. 3:**
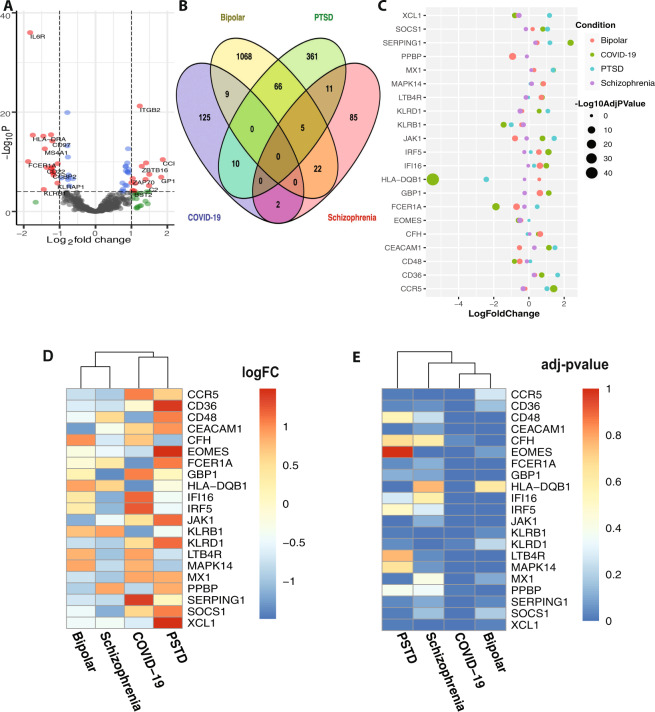
Targeted immune profiling of SARS Cov-2 infected patient blood using a Nanostring targeted immunology reveals systemic immune responses genes to infection, and shared common genes with the Bipolar disorder, PTSD and Schizophrenia. **A** Volcano plot of the Nanostring RNA-seq SARS-CoV-2 data shows genes with their threshold log fold changes and adjusted *p*-values to consider as significant genes. **B** Venn diagram shows the number of common significant genes of SARS Cov-2 in the immune panel with the bipolar disorder, PTSD and schizophrenia PBMC transcriptomic genes. **C** Bubble plot shows the combined log fold changes and adjusted *p*-values for the shared common genes between SARS-CoV-2 in the immune panel and either of the bipolar disorder, PTSD and schizophrenia PBMC transcriptomic genes. **D** Heat map of the log fold changes for the shared common genes between SARS-CoV-2 in the immune panel and either of the bipolar disorder, PTSD and schizophrenia. **E** Heat map of the adjusted *p*-values for the shared common genes between SARS-CoV-2 in the immune panel and either of the bipolar disorder, PTSD and schizophrenia, respectively, based on the PBMC transcriptomic analysis.

Thus far, we have extensively characterised the PBMC cell responses in COVID-19 patients that are also seen in patients with bipolar disorder, PTSD and schizophrenia. However, to understand how this may relate to the system-wide immune effects of COVID-19, we analysed data from a study of immune responses in PBMC of affected individuals. This study used a targeted immune panel on the NanoString platform which consisted of 759 immune genes. Compared to healthy controls, there were 145 significant genes altered in expression in COVID-19 patient samples, showing evidence of a distinct systemic infection response at the RNA level (Fig. 3A). We, therefore, compared these significant genes with the significant upregulated and downregulated genes seen in bipolar disorder, PTSD and schizophrenia, and found 9, 10 and 2 shared genes, respectively (Fig. 3B). Bubble and heat map plot visualisations showing the common transcriptional signature induced with COVID-19 and bipolar disorder, PTSD and schizophrenia are included in Fig. 3C–E. After establishing the common differential expression profiles associated with COVID-19 in the immune panel and in the three psychiatric disorders, we conducted a range of gene ontology and cell signalling pathway analyses. The top 25 significant signalling pathways and biological process ontology pathways for bipolar disorder, PTSD and schizophrenia, are presented in supplementary Fig. 2A–F, and all the significant pathways and GO are shown in the supplementary Tables 7–12.

### GWAS of COVID-19 infected patients revealed common biomarkers with data Mining result for bipolar, PTSD and schizophrenia conditions from the WGS and GWAS data

**Fig. 4:**
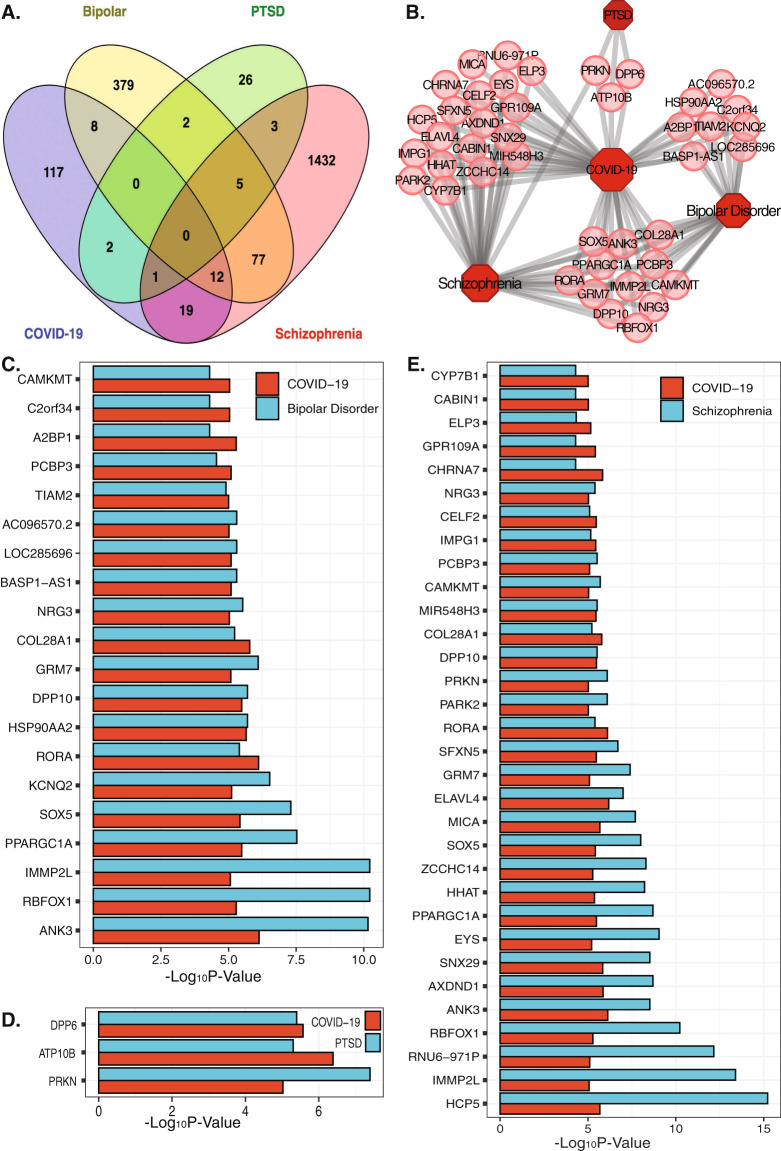
GWAS of SARS-CoV-2 infected patients reveal shared common significant genes with the bipolar disorder, PTSD and schizophrenia in GWAS and WGS datasets. **A** Venn diagram shows the number of common significant genes of SARS Cov-2 with the bipolar disorder, PTSD and schizophrenia genes. **B** Barchart shows the significant shared genes of SARS-CoV-2 and schizophrenia, and their corresponding 10 based −log *p*-values. **C** Barchart shows the significant shared genes of SARS-CoV-2 and bipolar disorder, and their corresponding 10 based −log *p*-values. **D** Barchart shows the significant shared genes of SARS-CoV-2 and PTSD, and their corresponding 10 based −log *p*-values. **E** Association network of the common significant genes identified in SARS-CoV-2 infected patients with the bipolar disorder, PTSD and schizophrenia in GWAS and WGS.

We have further analysed the GWAS data of COVID-19 from the https://www.covid19hg.org/results/. We found 146 genes related to COVID-19 status when considering *p*-values < 1.0E−6. We have also considered GWAS Catalogue^34^, GWAS ATLAS^35^, UK-Biobank^36^, dbGaP^37^, PheGenI^38^, and Clinver (https://www.ncbi.nlm.nih.gov/clinvar/) GWAS and WGS databases to identify the genome-wide significant genes for bipolar disorder, PTSD, and schizophrenia. We then cross-compared these selected marker genes from the GWAS and WGS databases with the 146 significant genes identified in the COVID-19 GWAS studies. Among these 146 significant genes, 20 genes are associated with bipolar disorder, 3 genes are associated with PTSD and 32 genes are associated with schizophrenia (Fig. 4A, B). It is notable that among these 32 genes one is found in common with the PTSD associated genes and 12 in common with the bipolar disorder-associated genes as shown in the network in Fig. 4B. Figure 4C–E show the significance levels of the common gene candidates from the GWAS and WGS studies. We also observed that only a few common genes are influenced by COVID-19 status for these three common psychiatric disorders. We performed signalling pathways and biological process gene ontology pathway analysis for these genes. The top 25 significant signalling pathways and ontology pathways are presented in supplementary Fig. 3A–F, and all the significant pathways are shown in the supplementary Tables 13–18.

### Disease-disease genetic correlations

Derived from the transcriptomic data based on PBMCs, we discovered that there are 39 genes shared by COVID-19 and bipolar disorder, 19 genes shared by COVID-19 and PTSD, and 22 genes shared by COVID-19 and schizophrenia. The hypergeometric test results show that both bipolar disorder (adjusted *p* = 2.5 × 10^−8^) and schizophrenia (adjusted *p* = 1.18 × 10^−52^) were significantly associated with COVID-19 at the genetic level (supplementary Table 19A). However, PTSD was not statistically significantly associated with COVID-19 (adjusted *p* > 0.05). When the candidate genes for COVID-19 were determined using the immune panel, we discovered that there are 9 genes shared by COVID-19 and bipolar disorder, 10 genes shared by COVID-19 and PTSD, and 2 genes shared by COVID-19 and schizophrenia. The hypergeometric analysis results suggest that both schizophrenia (adjusted *p* = 3.16 × 10^−8^) and PTSD (adjusted *p* = 2.25 × 10^−9^) were statistically significantly associated with COVID-19 profile (supplementary Table 19A). However, bipolar disorder was not statistically significantly associated with COVID-19 profile. Using data derived from GWAS studies, we discovered 20 genes shared by COVID-19 and bipolar disorder, 3 genes shared by COVID-19 and PTSD, and 32 genes shared by COVID-19 and schizophrenia. The hypergeometric analysis results suggest that both schizophrenia (adjusted *p* = 3.69 × 10^−7^) and PTSD (adjusted *p* = 0.0004) were statistically significantly associated with COVID-19. However, bipolar disorder was not statistically significantly associated with COVID-19 (supplementary Table 19A). The results suggest that schizophrenia is consistently associated with COVID-19 at the level of DNA variants and transcriptomic profiles. The correlation between PTSD and COVID-19 appears to be most prominent when the comparison is focused on the immune panel genes. However, the correlation between bipolar disorder and COVID-19 becomes nominal when the comparison is focused on the immune panel genes. On the contrary, the correlation between PTSD and COVID-19 is weak when the comparison is focused on genetic variants, while the correlation between bipolar disorder and COVID-19 remains significant when the comparison is focused on genetic variants.

The correlations between COVID-19 and the three psychiatric disorders at the pathway level are presented in supplementary Table 19B. The results indicate that PTSD is highly correlated with COVID-19 (hypergeometric *p*-value = 8.64 × 10^−140^ and Jaccard index value = 0.14 based on the whole-blood transcriptomic data; hypergeometric *p*-value = 8.64 × 10^−192^ and Jaccard index value = 0.18 based on the immune panel data). However, there was no overlapped pathway between COVID-19 and other psychiatric diseases based on the GWAS results. The results suggest that the shared genetic pathways between PTSD and COVID-19 could be considered to have a larger role in PTSD, compared with the role of the shared genetic pathways between bipolar disorder and COVID-19 or the role of the shared genetic pathways between schizophrenia and COVID-19 (supplementary Fig. 4).

### Co-expression and cluster analyses of the significant genes shared by COVID-19 with psychiatric disorders reveal that COVID-19 results in perturbation of expression of some genes that influence bipolar disorder, PTSD and schizophrenia

**Fig. 5:**
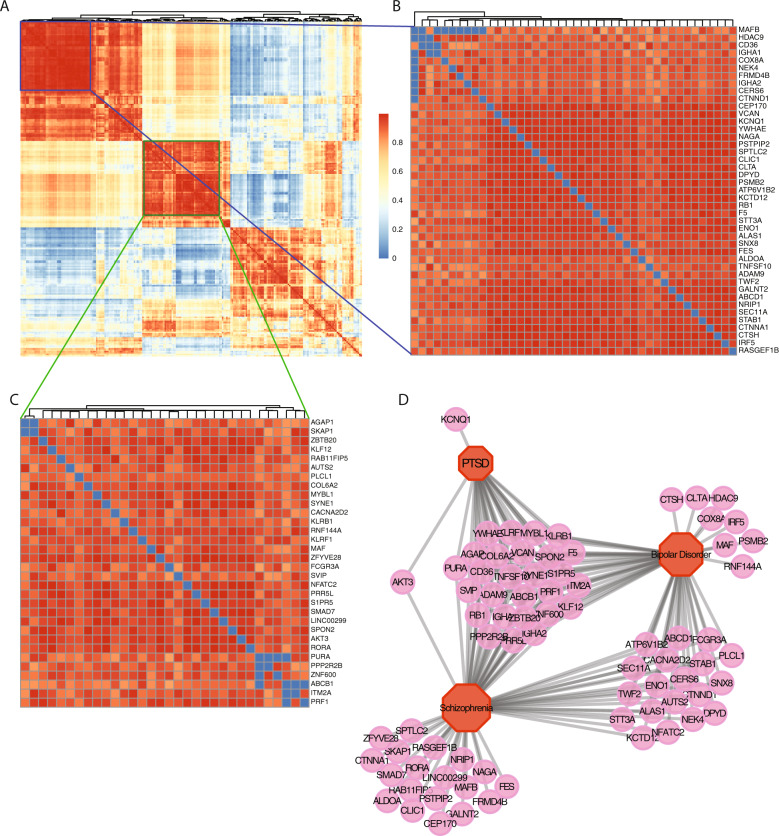
Coexpression and clustering analyses. **A** Coexpression and clustering analyses of the significant shared genes of SARS-CoV-2 with the bipolar disorder, PTSD and schizophrenia, reveal that SARS-CoV-2 infection could perturb some genes that are also affected in bipolar disorder, PTSD, and schizophrenia. Two clusters were selected based on the co-expression values in all the transcriptome samples used in this study. **B** One cluster is explored based on the correlation values>0.8 and it contains 43 genes that are coexpressed together. **C** The second cluster is also explored based on the correlation values>0.8 and it contains 32 genes that are coexpressed together. **D** The network of the genes of these two co-expressed clusters that shows some genes influenced by the SARS-CoV-2 are concordant in bipolar, PTSD, and schizophrenia.

For the co-expression analysis, we considered all the PBMC transcriptomic data in this study and all the significant genes of COVID-19 that are shared with the significant genes in the transcriptome, WGS and GWAS data of bipolar disorder, PTSD and schizophrenia. Figure 5A shows the correlation of all the shared genes. Hence we found that there are 2 clusters in which genes are very strongly coexpressed. We have highlighted the gene correlations of those 2 clusters with the correlation values > 0.8 in Fig. 5B, C. These two clusters contain 43 and 32 genes, respectively that are coexpressed together. Finally, we found that these 2 clusters shared significant genes between bipolar disorder, PTSD and schizophrenia for the SARS-CoV-2 infection as shown in Fig. 5D. Thus we observed that the network of the genes of these two co-expressed clusters showed that some genes influenced by COVID-19 status are concordant in psychiatric disorders. Thus the co-expression and clustering analyses of the genes with shared dysregulation between COVID-19 and bipolar disorder, PTSD, and schizophrenia, respectively, revealed that COVID-19 perturbates expression of some genes that are also implicated in these disorders. It is noteworthy that the proportion of the shared genes in PTSD appeared to be the largest among the three disorders studied.

## Discussion

COVID-19 has been demonstrated to affect many systems of the body, although it is highly controversial as to whether it affects the brain directly to influence human behaviour. It is nevertheless clear that SARS-CoV-2 can affect neuronal functions and can result in neuropsychiatric symptoms^39^. This raises the possibility that there could be COVID-19 interactions on psychiatric disorders due to direct infection of CNS neurons or indirectly due to infection of cells of the immune system or other mediating systems such as the HPA axis. There is some circumstantial evidence for COVID-19 interaction with psychiatric disorders and these observations could be attributed to the psychological stress (due to the pandemic situation) or through stress system responses. It is clear that the SARS-CoV-2 has an impact on the immune system involved in psychiatric comorbidities, although it is difficult to confirm without brain tissue samples^40^. Nevertheless, since immune dysregulation may play a role in psychiatric diseases, transcriptomic profiles of PBMCs associated with immune responses may still provide valuable clues to genetic functional changes in psychiatric disorders that are shared with COVID-19 infection. In addition, previous evidence suggests that a proportion of gene expressions in PBMCs are correlated with gene expressions in brain tissues. For example, approximately a half of the candidate genes of schizophrenia are expressed in both the whole blood and the prefrontal cortex; and their expression levels are not significantly different between the whole blood and the prefrontal cortex^41^.

In this study, we have for the first time performed extensive transcriptomic characterisation of the effects of the COVID-19 in comparison to three psychiatric disorders namely bipolar disorder, PTSD and schizophrenia. To our knowledge, this is the largest aggregate comparative genomic and transcriptomic study of patients infected with the novel coronavirus and compared with psychiatric disorders. Our findings suggest that inflammatory signalling pathways dominate the acute COVID-19 infection response, and while there is little concordance at the level of individual genes between responses of PBMCs, there is concordance seen between COVID-19 and psychiatric disorders in a set of perturbed cell signalling pathways. This might be expected if the PBMC responses are indirect rather than due to direct results of blood cell SARS-CoV-2 infection. These observations may hence prove therapeutically relevant as normalising some of these pathways might ameliorate the COVID-19 disease progression through some of the pathways that are activated in newly infected PBMCs. Although the pathogenic role of these pathways is not clear, inflammation blockade could be beneficial for COVID-19 patients. Further analyses of clinical data will be needed to clarify this.

We also found that the disease-disease correlations depend on the type of studies (i.e., transcriptomic versus GWAS results), as well as how we measure overlapped genetic networks. When the candidate genes are derived from GWAS studies, both COVID-19 and PTSD have fewer genes than bipolar disorder or schizophrenia. This may be attributable to the fact that both COVID-19 and PTSD are pathological responses to a pathogen and psychological stressor, respectively, and hence the signals of genomic functional changes that respond to these environmental triggers would be more detectable than the signals for effects of DNA variants on such phenotypes. As a result, the correlation between COVID-19 and PTSD based on the GWAS findings might not reflect genuine genomic similarities shared by these two diseases. Interestingly, transcriptomic analysis results show that PTSD and COVID-19 have a stronger correlation based on the overlapped genes when we focused on immune-related genes compared with the gene set extracted from the whole genome. PTSD and COVID-19 appear to show a higher level of correlation based on shared GO pathways than the correlation between bipolar disorder and COVID-19 and the correlation between schizophrenia and COVID-19. Therefore, the pathway-based analyses have lent support to the hypothesis that the role of stress in genomic functional changes in PTSD may play, to a certain degree, a similar role in genomic functional changes in COVID-19. The shared genomic functional changes between COVID-19 and all these three psychiatric disorders involve proinflammatory responses and cytokine secretion. Specifically, in the case of PTSD where patients are prone to the development of a pro-inflammatory state, additional exposure to infections such as COVID-19 that trigger autoimmune and inflammatory responses may lead to exaggerated or unusual disease progression and outcomes. Interactive effects between stress, HPA axis, and cytokines, are well established by clinical and preclinical studies, which indicate that exposure to stress induces increased levels of pro-inflammatory cytokines and reactive oxygen species in the brain (hippocampus, amygdala, pre-frontal cortex) and in the periphery^42^. Further, it has been shown that the administration of anti-inflammatory agents such as minocycline following exposure to a stressor can block the development of PTSD-like behaviours in rodent models^43^. Given the mediating effect of chronic inflammation in PTSD and conditions such as cardiovascular disease and chronic respiratory conditions that increase the risk for COVID-19 infection, a better understanding of such disease interaction pathways might open up opportunities for therapeutic interventions. Drug repositioning based on shared genomic functional changes, such as pro-inflammatory responses, may hence hold the key to novel treatment options that can substantially reduce comorbid symptoms for patients that suffer from COVID-19 and co-occurring psychiatric illnesses.

## Conclusions

We performed RNA-Seq, GWAS and data mining analyses to compare the infection responses of SARS-CoV-2 to the progression of the bipolar disorder, PTSD and schizophrenia. We found COVID-19 patients to show a distinctive transcriptional profile in PMBCs, dominated by inflammatory cytokine and interferon response genes, consistent with a pro-inflammatory state which may affect bipolar disorder, PTSD, and schizophrenia. These findings increase our understating of the association of COVID-19 with these psychiatric disorders through the cytokine and interferon response genes and show how the infection might be examined for other diseases. A better understanding of how shared pro-inflammatory responses and cytokine regulation pathways may contribute to the increased risk of COVID-19 in psychiatric patients may shed light on novel therapeutic targets. Thus we could develop some therapeutic drugs for SARS-CoV-2 infected psychiatric patients.

## Materials and methods

### Data Preprocessing and Identification of differentially expressed genes

We analysed two COVID-19 related RNA-Seq transcriptomic datasets. One was derived from a study of PBMC from patients infected with SARS-CoV-2 from the Beijing Institute of Genomics Genome Sequence Archive in BIG Data Center (https://bigd.big.ac.cn/), P.R. China, data accession number: CRA002390. This was a study of 3 infected patients and 3 healthy controls from Zhongnan Hospital of Wuhan University, P.R. China, using RNA extracted from PBMC derived from 4 ml of peripheral blood that was reverse transcribed and sequenced with an MGISEQ-2000 platform (MGI, Shenzhen, P.R. China)^44^. The second dataset was from a study of immune responses in healthy controls and COVID-19 cases that employed a NanoString Human Immunology Panel to profile collected peripheral blood cells RNA extracted from whole blood samples (E-MTAB-8871)^45^. This was a profile of the transcriptomes of PBMCs extracted from 3 SARS-CoV-2 infected individuals. Blood samples were taken daily, for up to 19 days, then RNA extracted analysed by Nanostring sequencing. This analysis differed from the above PBMC study in that prior to sequencing PBMC RNA was processed to extract mRNA of a defined panel of an inflammatory panel by a hybridisation based method with nCounter Sprint Profiler^46^.

In addition to the above COVID-19 studies, we also analysed transcriptomic data for the bipolar disorder (NCBI GEO accession GSE46449) using Affymetrix Human Genome U133 Plus 2.0 Array. This dataset employed RNA from PBMCs from blood samples from 26 medicated bipolar disorder patients and 25 matched healthy control individuals^47^. PTSD patient PBMCs were studied (NCBI GEO accession GSE860) using the Affymetrix Human Genome U95A Array. Samples were taken in the hospital emergency department within hours of experiencing severe trauma, and four months later; some of the patients developed chronic PTSD (17 samples) and others recovered and were employed as a control group (16 samples)^48^. To study PBMC transcript profiles for schizophrenia patients we employed the GEO accession GSE27383 dataset which used Affymetrix Human Genome U133 Plus 2.0 arrays^49^. This study included 43 affected patients with 28 matched controls. All of these transcriptomic data were generated from the PBMCs. We used the DESeq2 R Bioconductor package to analyse all these RNA-Seq transcriptomic data and the LIMMA (linear models for microarray data) R Bioconductor package was used to analyse gene expression data sets. We removed the batch effect using the quintile normalisation method. An adjusted *p*-value <0.05 and the absolute value of log2 fold change (LFC) ≥1 were regarded as threshold criteria to define significant differentially expressed genes (DEGs) of interest. We used the Benjamini–Hochberg (BH) method to control the false discovery rate to adjust for multiple tests.

### GWAS and data mining to identify stablished gene markers in GWAS and WGS studies

We have utilised meta-analyses data from the COVID-19 GWS consortium (https://www.covid19hg.org/results/). This large meta-analysis examined GWAS data (i.e., the incidence of variants in genomic DNA) from ill and hospitalised COVID-19 patients. This data was gathered to identify SNPs and other types of DNA variants that influenced the development of severe symptoms in infected patients. After analysing this data we found 146 genes related to COVID-19 status, after considering the *p*-value threshold of <1.0E−6. We also considered GWAS Catalogue, GWAS ATLAS, UK-Biobank, dbGaP, PheGenI and Clinver GWAS and WGS databases to identify the genome-wide significant genes associated with the development of the bipolar disorder, PTSD and schizophrenia. Then we cross-compared these candidate genes from the GWAS and WGS databases with differentially expressed genes identified in the SARS-CoV-2 transcriptome studies.

### Gene ontology and cell signalling pathway analyses

We performed gene set enrichment analysis of Gene Ontology and cell signalling pathways to evaluate the biological relevance and functional pathways of the shared significant genes between SARS-CoV-2 and each of the bipolar disorder, PTSD and schizophrenia. All functional analyses were performed using the Enrichr {https://amp.pharm.mssm.edu/Enrichr/} software tools^50^. For cell signalling pathway enrichment analyses we employed KEGG^51^, WikiPathways^52^, BioCarta^53^ and Reactome^54^, databases. We used the GO Biological Process (2018) database for gene ontological analysis^55^. In this work an adjusted *P*-value ≤0.05 was considered as statistically significant for enrichment analysis.

For each gene set of our interest from the selected pathways and GO terms, we calculated the frequency (*f*) of genes in the study set (*s*) that interact with a pathway, and the frequency (*F*) of genes in the population set (*S*) that interact with the same pathway. We then performed a test to determine how likely it would be to obtain at least *f* genes interacting with a pathway if *s* genes would be randomly drawn from the population, given that the frequency *F* and size *S* of the population. This can be represented mathematically as follows:

$$P\left( f \right) = \frac{{\left( {\frac{F}{f}} \right)\left( {\frac{{S - s}}{{F - f}}} \right)}}{{\frac{S}{s}}}$$

### Disease-disease genetic correlations

We further examined the contribution of the genes significantly associated with COVID-19 that were also associated with each psychiatric disorder. The probability of finding significant disease-disease associations by random chance was calculated using the hypergeometric distribution. The mean of the distribution is equal to *n* × *k*/*N*. The variance is *n* × *k* × (*N* − *k*) × (*N* − *n*)/[*N*^2^ × (*N* − 1)], where *N*, *k*, *n* and *x*, denote the numbers of the genes in the whole genome, the number of the genes associated with the psychiatric disorder, the number of genes associated with COVID-19, and the number of candidate genes shared by both the particular psychiatric disorder and COVID-19. This probability was used to determine the level of disease-disease correlation at the gene level. The *p*-values were adjusted using the Benjamini–Hochberg procedure for multiple-testing corrections. We also compared the overlapped pathways between COVID-19 using the same formula, while *N*, *k*, *n* and *x*, denote the numbers of all GO terms, the number of the GO pathways associated with the psychiatric disorder, the number of GO pathways associated with COVID-19, and the number of GO pathways shared by both the psychiatric disorder and COVID-19. Furthermore, we calculated the Jaccard index value^56^ to examine the disease-disease correlation at the pathway level. Jaccard index can be expressed as C/(A + B − C), where A, B and C, denote the number of pathways associated with COVID-19, the psychiatric disorder, and the number of pathways shared by both COVID-19 and the psychiatric disorder.

### Co-expression analysis and cluster analysis

At first, we have selected all the identified significant genes of COVID-19 that shared with the significant genes in any of the transcriptomic, WGS and GWAS data of bipolar disorder, PTSD and schizophrenia to identify the co-expression and cluster analysis. Considering these selected genes we have identified their corresponding PBMC transcriptomic expression data that were used in the whole study for different conditions. Then we have used Pearson correlation for all these genes towards all the samples in different conditions. Then we applied the hierarchical clustering to this co-expression data and identified two most significant clusters (with the correlation values >0.8) in which genes are very strongly coexpressed. Then considering the shared significant genes between bipolar disorder, PTSD, and schizophrenia for the SARS-CoV-2 infection that exists in these 2 clusters we built a gene-disease network using the Cytoscape^57^.

## Supplementary information

Supplementary Figure Captions

Supplementary Figure 1

Supplementary Figure 2

Supplementary Figure 3

Supplementary Figure 4

Supplementary Table 1-6

Supplementary Table 7-12

Supplementary Table 13-18

Supplementary Table 19

## Data Availability

All analyses results and programing codes can be freely accessed through the GitHub repository at: https://github.com/m-moni/COVID-19.
