# Supplementary material for: COVID-19 patient transcriptomic and genomic profiling reveals comorbidity interactions with psychiatric disorders
Source: Transl Psychiatry. 2021 Mar 15;11:160. doi: 10.1038/s41398-020-01151-3 (PMC7957287; doi:10.1038/s41398-020-01151-3)
Supplement: Supplementary file 1 — Supplementary Figure Captions [file 41398_2020_1151_MOESM1_ESM.docx]

**Supplementary Figure Captions:**

Supplementary FIGURE 1: Top cell signalling pathways and Gene ontology analyses find enriched in whole PBMC. A-B) Signaling pathways and Gene ontology analyses enriched with the shared significant genes between SARS-Cov-2 infection and Bipolar disorder. C-D) Signaling pathways and Gene ontology analyses enriched with the shared significant genes between SARS-Cov-2 infection and PTSD. E-F) Signaling pathways and Gene ontology analyses enriched with the shared significant genes between SARS-Cov-2 infection and Schizophrenia. Analyses performed using The Gene Ontology, WikiPathways, BioCarta, Reactome, and Panther databases.

Supplementary FIGURE 2: Top cell signalling pathways and Gene ontology analyses find enriched in the immune panel. A-B) Signaling pathways and Gene ontology analyses enriched with the shared significant genes between SARS-Cov-2 infection in the immune panel and Bipolar disorder. C-D) Signaling pathways and Gene ontology analyses enriched with the shared significant genes between SARS-Cov-2 infection in the immune panel and PTSD. E-F) Signaling pathways and Gene ontology analyses enriched with the shared significant genes between SARS-Cov-2 infection in the immune panel and Schizophrenia. Analyses performed using The Gene Ontology, WikiPathways, BioCarta, Reactome, and Panther databases.

Supplementary FIGURE 3: Top cell signalling pathways and Gene ontology analyses find enriched in GWAS and WGS. A) Signaling pathways and Gene ontology analyses enriched with the shared significant genes between SARS-Cov-2 infection and Bipolar disorder. B) Signaling pathways and Gene ontology analyses enriched with the shared significant genes between SARS-Cov-2 infection and PTSD. C. Signaling pathways and Gene ontology analyses enriched with the shared significant genes between SARS-Cov-2 infection and Schizophrenia. Analyses performed using The Gene Ontology, WikiPathways, BioCarta, Reactome, and Panther databases.

Supplementary FIGURE 4: Correlation between COVID-19 and three psychiatric disorders. Jaccard index and Hypergeometric test values between COVID-19 with the bipolar disorder, PTSD and schizophrenia are shown.
