# Supplementary figures and images for: COVID-19 patient transcriptomic and genomic profiling reveals comorbidity interactions with psychiatric disorders
Source: Transl Psychiatry. 2021 Mar 15;11:160. doi: 10.1038/s41398-020-01151-3 (PMC7957287; doi:10.1038/s41398-020-01151-3)

Supplementary Figure 1

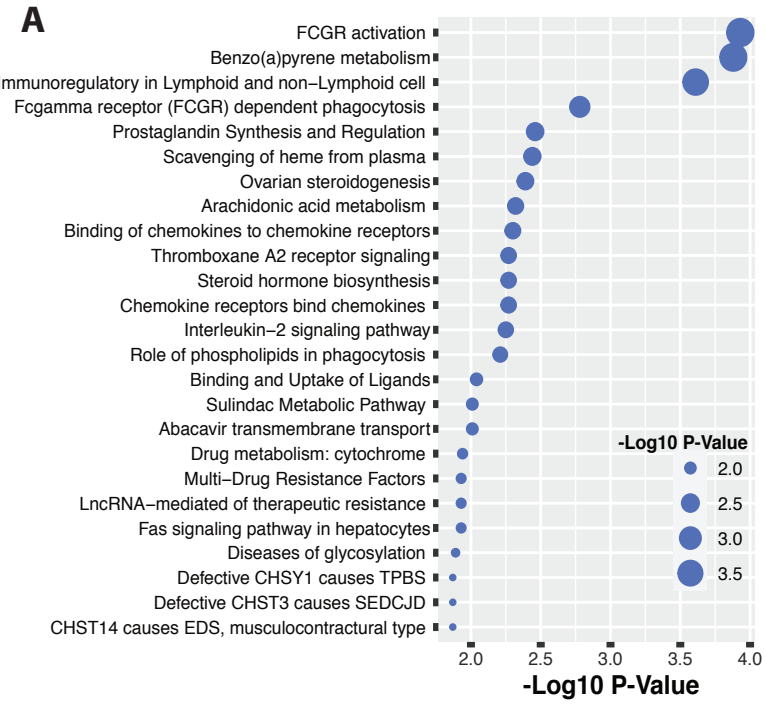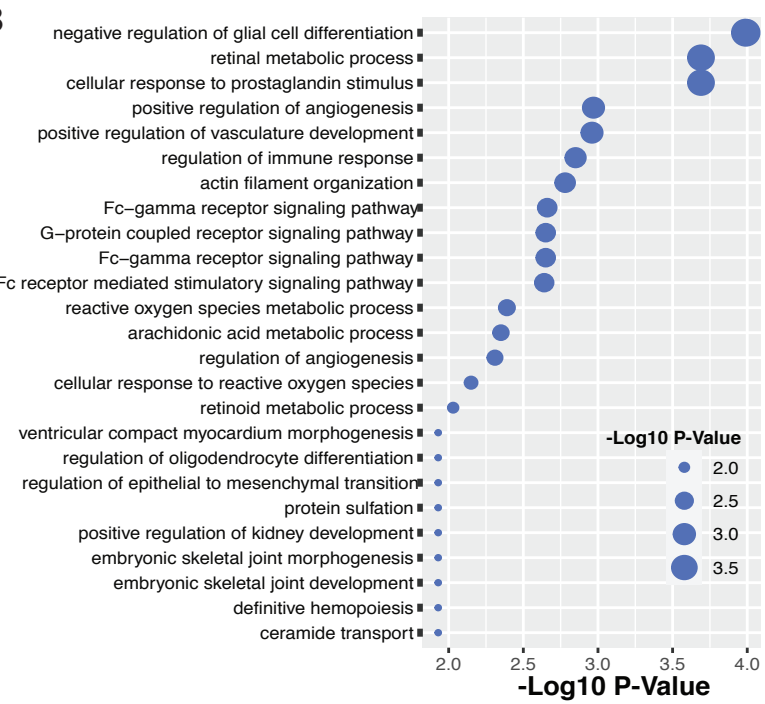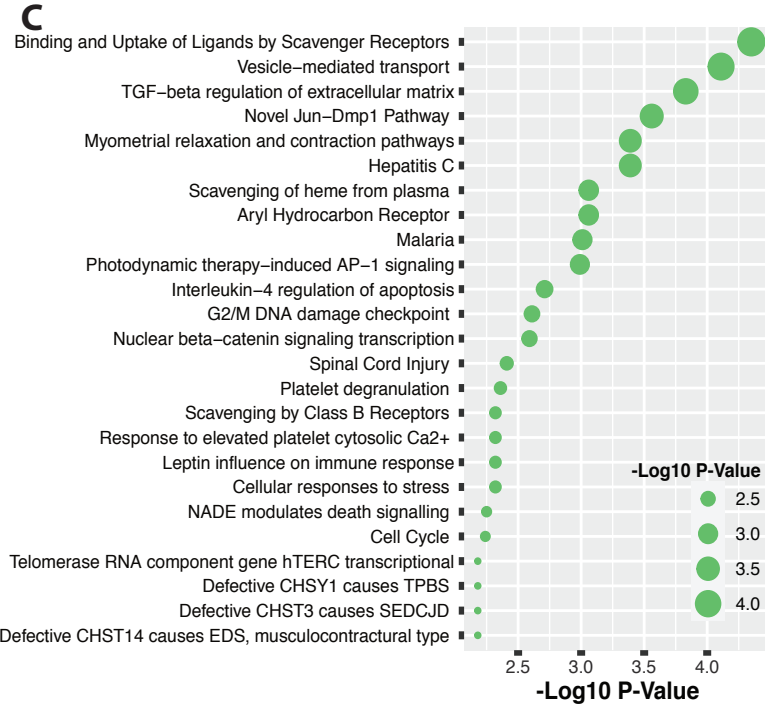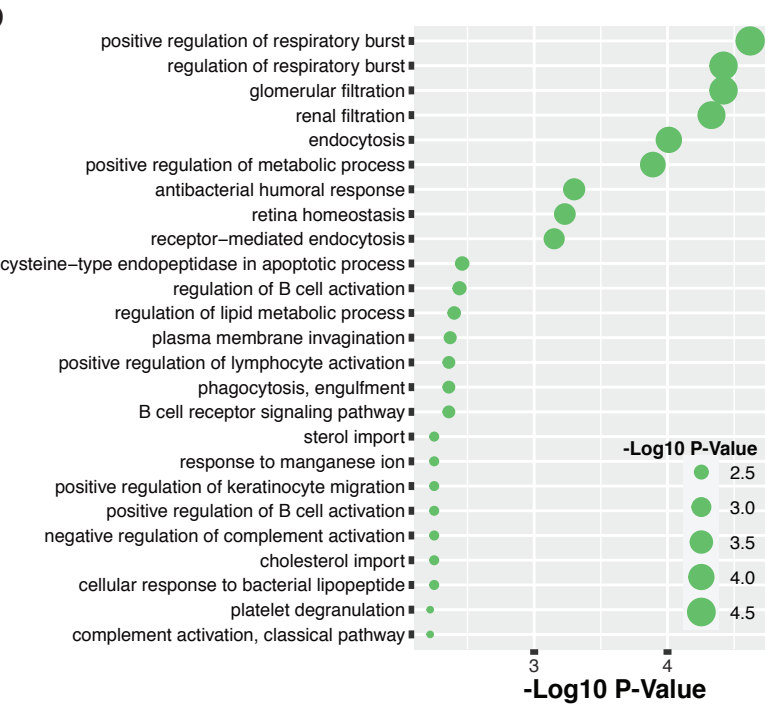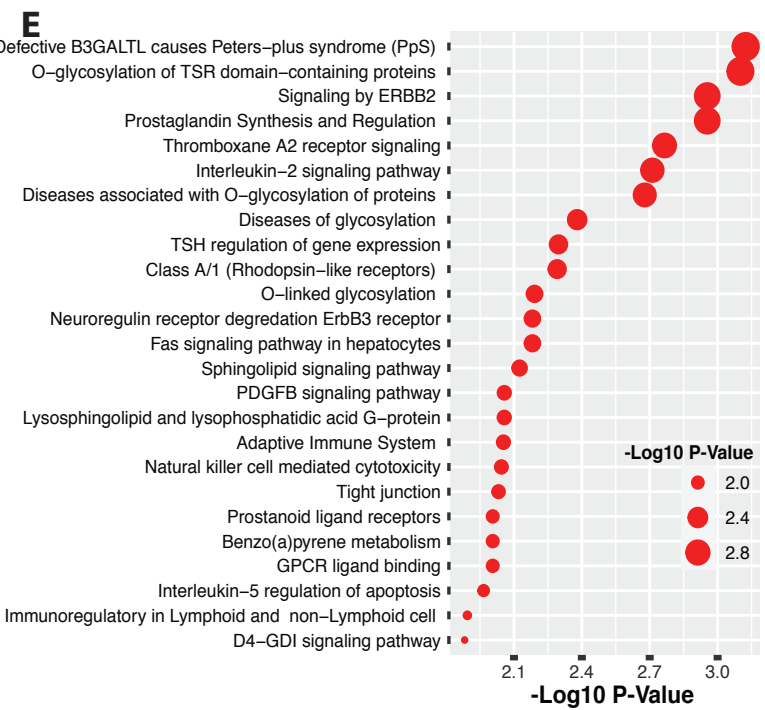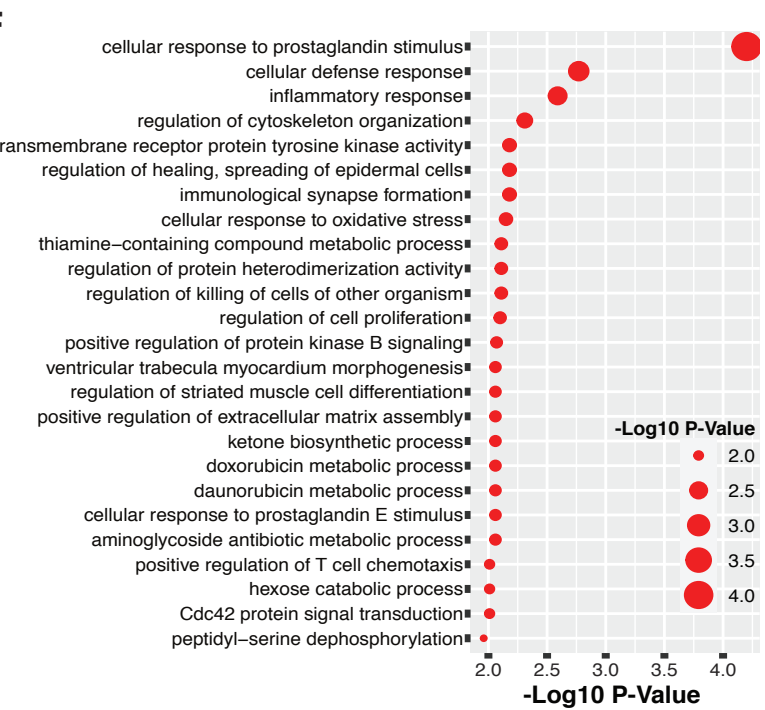

Supplement: Supplementary file 2 — Supplementary Figure 1 [file 41398_2020_1151_MOESM2_ESM.pdf]

# Supplementary Figure 2

**A**

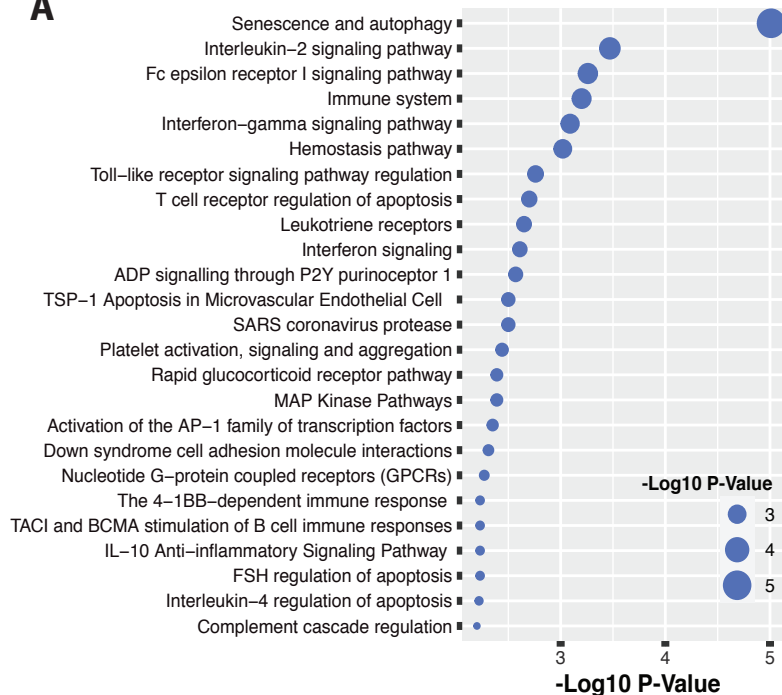

**B**

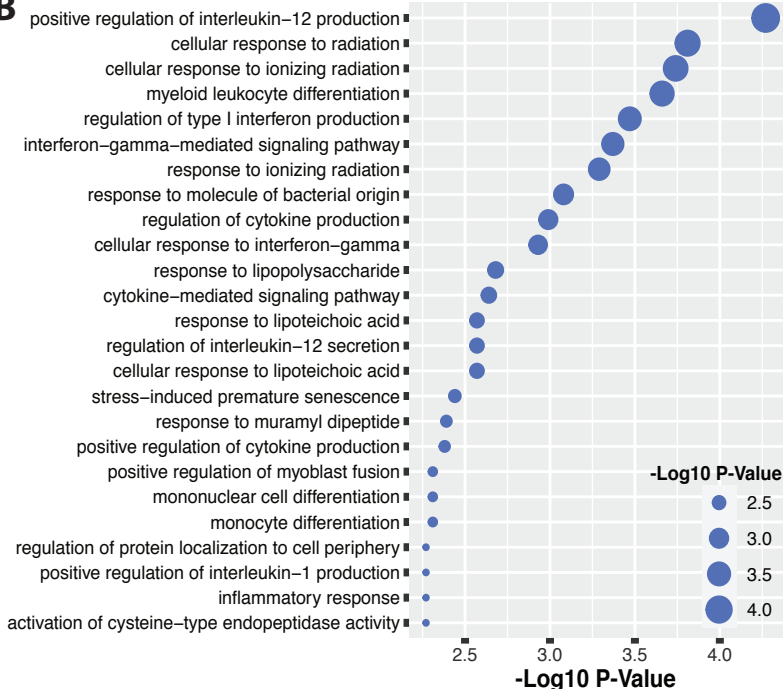

**C**

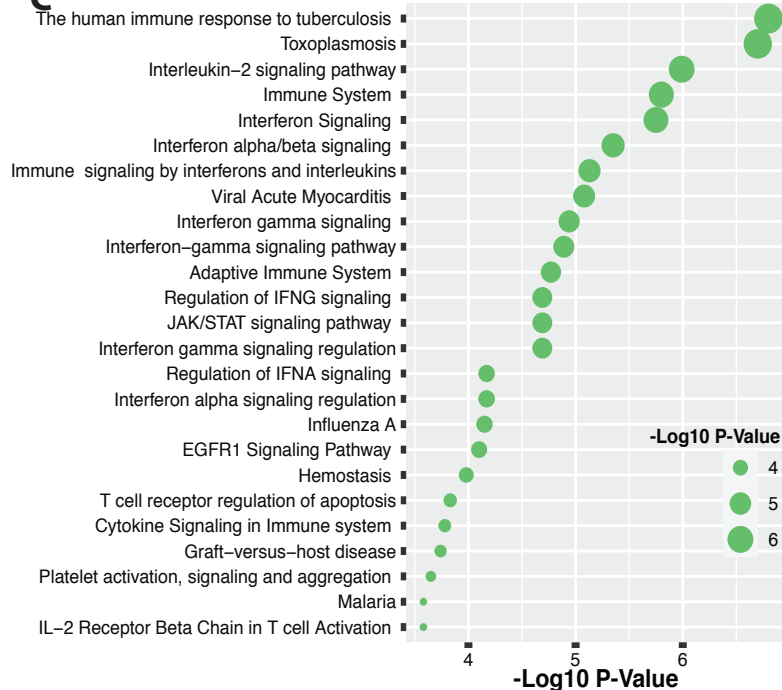

**D**

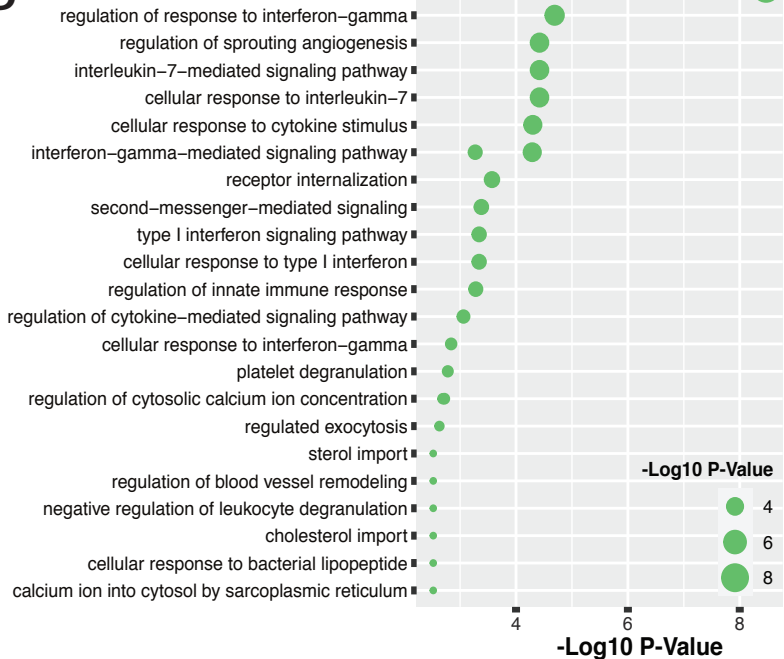

**E**

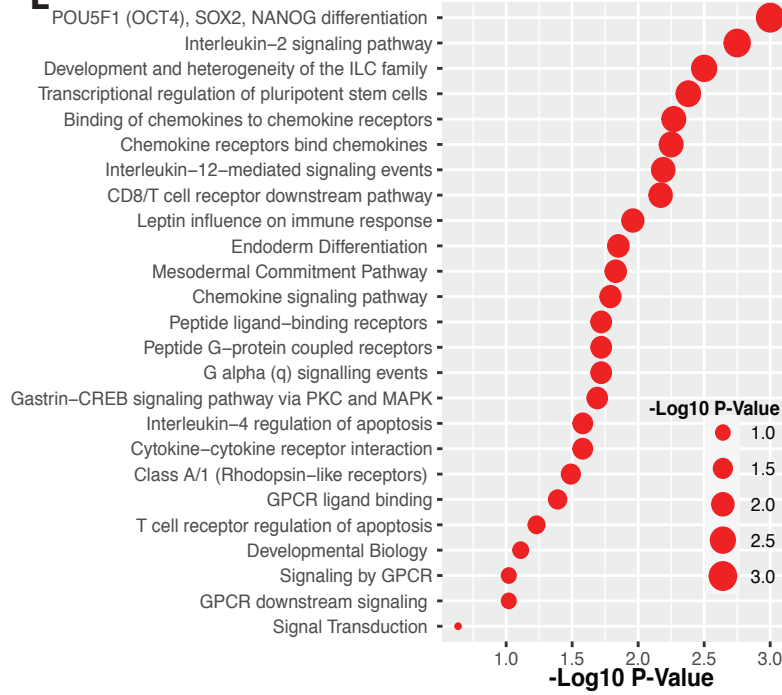

**F**

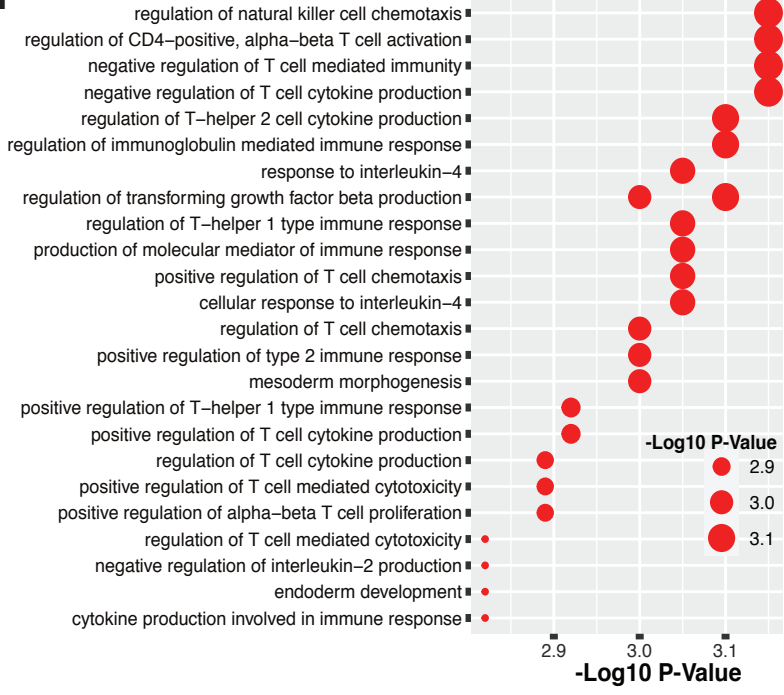

Supplement: Supplementary file 3 — Supplementary Figure 2 [file 41398_2020_1151_MOESM3_ESM.pdf]

# Supplementary Figure 3

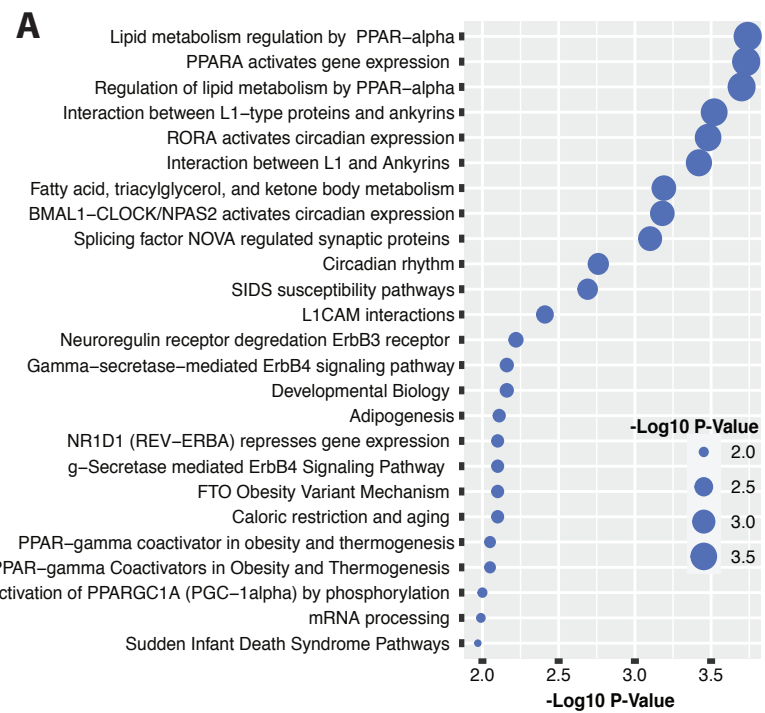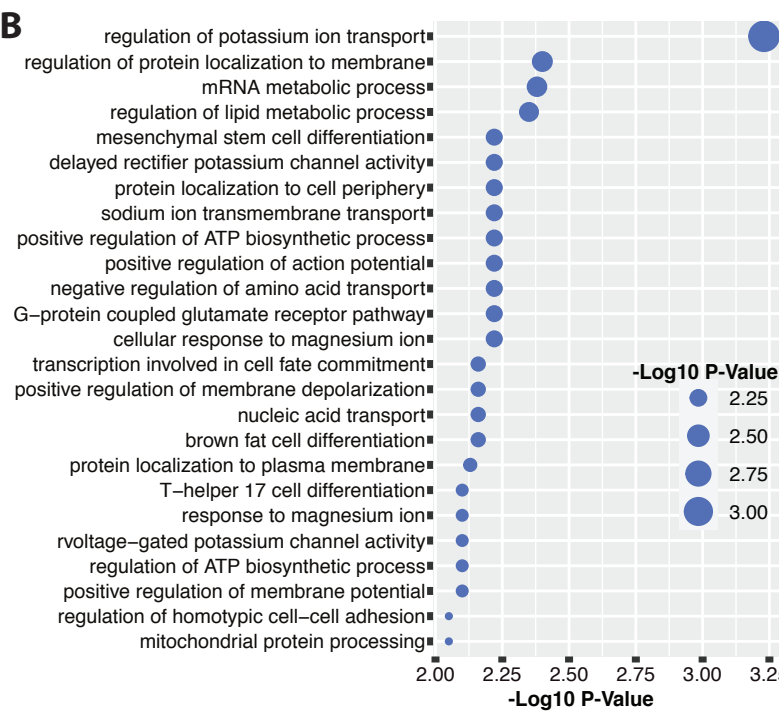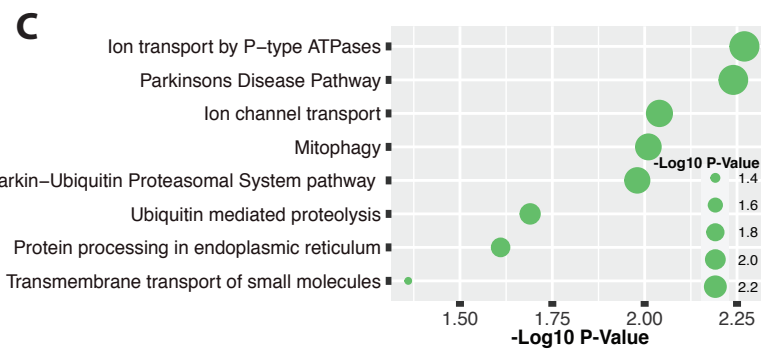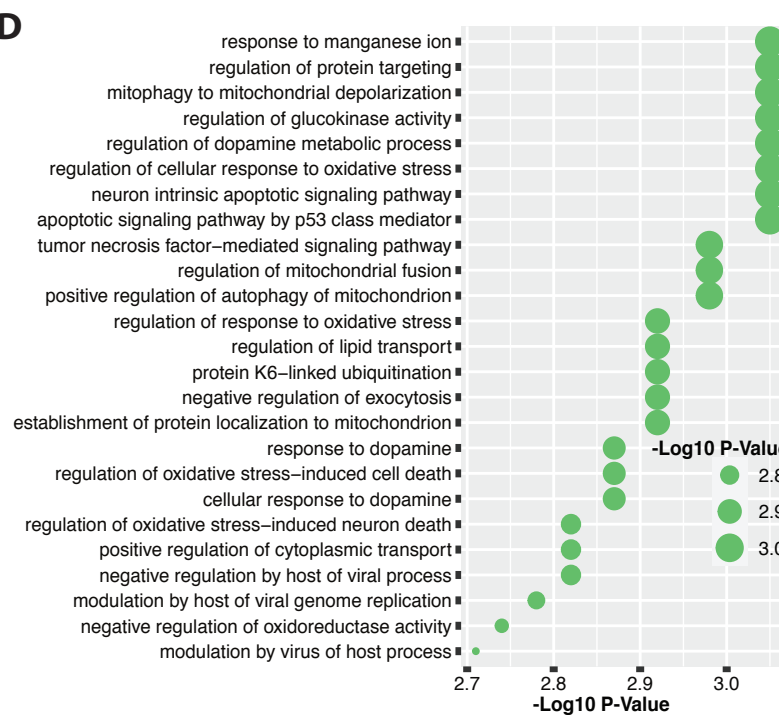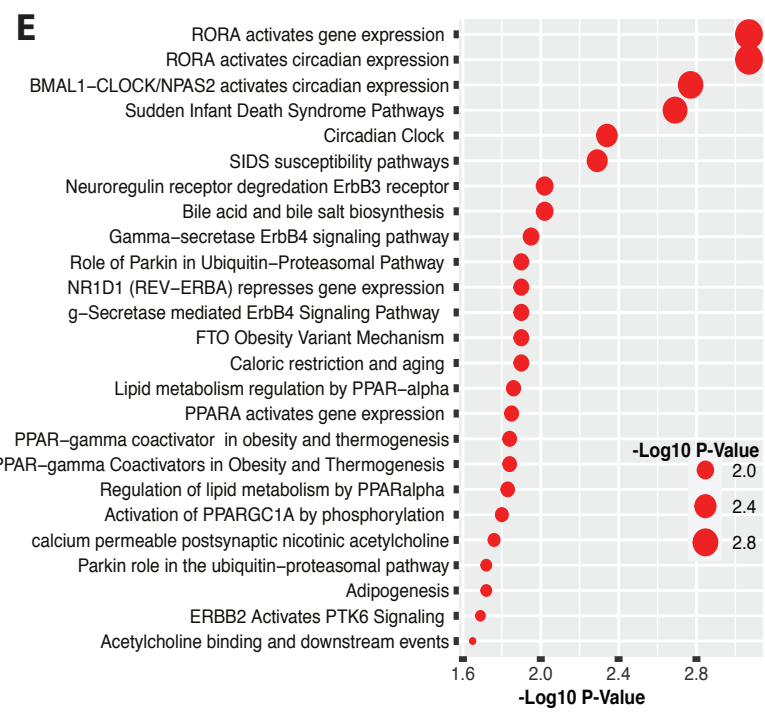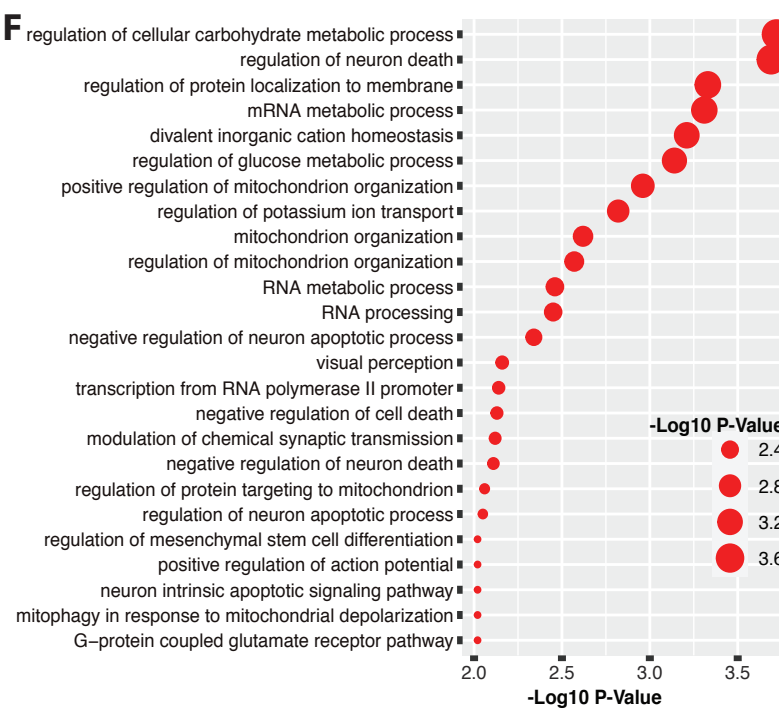

Supplement: Supplementary file 4 — Supplementary Figure 3 [file 41398_2020_1151_MOESM4_ESM.pdf]

Supplementary Figure 4

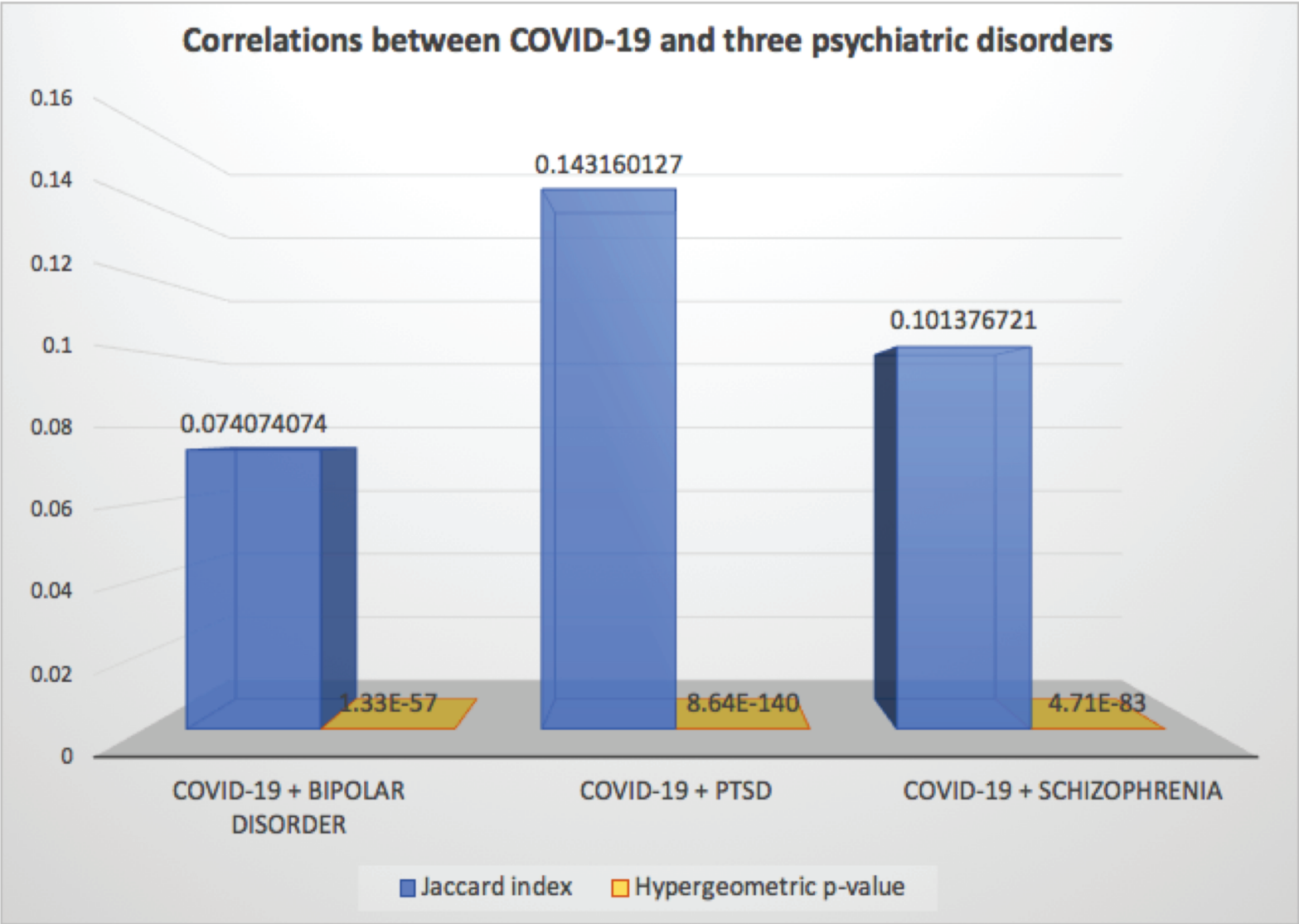

Supplement: Supplementary file 5 — Supplementary Figure 4 [file 41398_2020_1151_MOESM5_ESM.pdf]
