# Supplementary material for: COVID-19 patient transcriptomic and genomic profiling reveals comorbidity interactions with psychiatric disorders
Source: Transl Psychiatry. 2021 Mar 15;11:160. doi: 10.1038/s41398-020-01151-3 (PMC7957287; doi:10.1038/s41398-020-01151-3)
Supplement: Supplementary file 9 — Supplementary Table 19 [file 41398_2020_1151_MOESM9_ESM.docx]

**Supplementary Table 19A**. Disease-disease correlations assessed using the hypergeometric distributions at the gene level.

| **PBMC** |  |  |
| --- | --- | --- |
|  | **Unadjusted p-value** | **H-B adjusted p-value^*^** |
| COVID-19 and schizophrenia | 3.94E-53 | 1.18E-52 |
| COVID-19 and bipolar disorder | 1.25E-08 | 2.50E-08 |
| COVID-19 and PTSD | 6.49E-02 | 6.49E-02 |
|  |  |  |
| **Immune panel** |  |  |
|  | **Unadjusted p-value** | **H-B adjusted p-value^*^** |
| COVID-19 and schizophrenia | 1.58E-08 | 3.16E-08 |
| COVID-19 and bipolar disorder | 8.80E-02 | 8.80E-02 |
| COVID-19 and PTSD | 7.49E-10 | 2.25E-09 |
|  |  |  |
| **GWAS** |  |  |
|  | **Unadjusted p-value** | **H-B adjusted p-value^*^** |
| COVID-19 and schizophrenia | 1.23E-07 | 3.69E-07 |
| COVID-19 and bipolar disorder | 1.38E-04 | 4.13E-04 |
| COVID-19 and PTSD | 8.35E-02 | 2.51E-01 |

*H-B indicates the Benjamini–Hochberg procedure.

**Supplementary Table 19B.** Disease-disease correlations assessed using hypergeometric distributions and Jaccard index at the pathway level are shown. GWAS-based data are not shown.

| **Whole blood** |  |  |  |  |  |  |
| --- | --- | --- | --- | --- | --- | --- |
| **Disease pair** | **A** | **B** | **C** | **Jaccard^*^ index** | **Hypergeometric p-value** | **-Log10(p-value)** |
| COVID-19 + bipolar disorder | 586 | 255 | 58 | 0.074 | 1.33E-57 | 56.88 |
| COVID-19 + PTSD | 586 | 492 | 135 | 0.143 | 8.64E-140 | 139.06 |
| COVID-19 + schizophrenia | 586 | 294 | 81 | 0.101 | 4.71E-83 | 82.33 |
|  |  |  |  |  |  |  |
| **Immune panel** |  |  |  |  |  |  |
| **Disease pair** | **A** | **B** | **C** | **Jaccard index** | **Hypergeometric p-value** | **-Log10(p-value)** |
| COVID-19 + bipolar disorder | 588 | 255 | 59 | 0.075 | 5.51E-59 | 58.26 |
| COVID-19 + PTSD | 588 | 492 | 167 | 0.183 | 2.43E-192 | 191.61 |
| COVID-19 + schizophrenia | 588 | 294 | 113 | 0.147 | 1.38E-135 | 134.86 |

* Jaccard index = C/(A+B-C)​
